# Fish intake and pre-frailty in Norwegian older adults - a prospective cohort study: the Tromsø Study 1994–2016

**DOI:** 10.1186/s12877-023-04081-z

**Published:** 2023-07-05

**Authors:** Dina Moxness Konglevoll, Lene Frost Andersen, Laila Arnesdatter Hopstock, Bjørn Heine Strand, Magne Thoresen, Torunn Holm Totland, Anette Hjartåker, Monica Hauger Carlsen

**Affiliations:** 1 Department of Nutrition, Institute of Basic Medical Sciences, University of Oslo, Oslo, Norway; 2 Department of Health and Care Sciences, UiT The Arctic University of Norway, Tromsø, Norway; 3 The Norwegian National Centre for Ageing and Health, Vestfold Hospital Trust, Tønsberg, Norway; 4 Department of Geriatric Medicine, Oslo University Hospital, Oslo, Norway; 5 Department of Physical Health and Ageing, Norwegian Institute of Public Health, Oslo, Norway; 6 Department of Biostatistics, Institute of Basic Medical Sciences, University of Oslo, Oslo, Norway

**Keywords:** Ageing, Diet, Epidemiology, Fish, Pre-frailty, Geriatrics

## Abstract

**Background:**

Pre-frailty is an intermediate, potentially reversible state before the onset of frailty. Healthy dietary choices may prevent pre-frailty. Fish is included in most healthy diets, but little is known about the association between long-term habitual fish intake and pre-frailty. We aimed to elucidate the longitudinal association between the frequency of fish intake and pre-frailty in a cohort of older adults in Norway.

**Methods:**

4350 participants (52% women, ≥65 years at follow-up) were included in this prospective cohort study. Data was obtained from three waves of the population-based Tromsø Study in Norway; Tromsø4 (1994–1995), Tromsø6 (2007–2008) and Tromsø7 (follow-up, 2015–2016). Frailty status at follow-up was defined by a modified version of Fried’s phenotype. Fish intake was self-reported in the three surveys and assessed as three levels of frequency of intake: low (0–3 times/month), medium (1–3 times/week) and high (≥ 4 times/week). The fish–pre-frailty association was analysed using multivariable logistic regression in two ways; (1) frequency of intake of lean, fatty and total fish in Tromsø6 and pre-frailty at follow-up, and (2) patterns of total fish intake across the three surveys and pre-frailty at follow-up.

**Results:**

At follow-up, 28% (*n* = 1124) were pre-frail. Participants with a higher frequency of lean, fatty and total fish intake had 28% (odds ratio (OR) = 0.72, 95% confidence interval (CI) = 0.53, 0.97), 37% (OR = 0.63, 95% CI = 0.43, 0.91) and 31% (OR = 0.69, 95% CI = 0.52, 0.91) lower odds of pre-frailty 8 years later compared with those with a low intake, respectively. A pattern of stable high fish intake over 21 years was associated with 41% (OR = 0.59, 95% CI = 0.38, 0.91) lower odds of pre-frailty compared with a stable low intake.

**Conclusions:**

A higher frequency of intake of lean, fatty and total fish, and a pattern of consistent frequent fish intake over time, were associated with lower odds of pre-frailty in older community-dwelling Norwegian adults. These results emphasise the important role of fish in a healthy diet and that a frequent fish intake should be promoted to facilitate healthy ageing.

**Supplementary Information:**

The online version contains supplementary material available at 10.1186/s12877-023-04081-z.

## Background

A key focus in ageing research is the frailty syndrome [1]. Frailty is a transitional state between healthy ageing and disability in older adults, and frailty prevention is significantly important at both societal and individual level [2]. Frail individuals are less resilient to trauma and stress and more prone to adverse outcomes than non-frail individuals of the same chronological age [3, 4].

Physical frailty has been defined by Fried et al. by the following five characteristics: exhaustion, unintentional weight loss, low physical activity, slowness and weakness [5]. The presence of three or more of these characteristics classifies individuals as frail, whereas the presence of one or two classifies individuals as pre-frail, an intermediate state with an elevated risk of progression to frailty [4–7]. Frailty is a dynamic syndrome and, therefore, pre-frailty and frailty are potentially reversible [6, 8]. The importance of early interventions has been emphasized and, specifically, the pre-frail state has been identified as a suitable target for preventive measures [4, 8].

Research suggests that there is an association between a healthy diet and lower risk of frailty in older adults [9–11]. The vast majority of existing studies focus on frailty rather than pre-frailty, but a recently published systematic review and meta-analysis found that a higher adherence to the Mediterranean diet [12] was associated with lower risk of pre-frailty [13]. Fish is a food group that is often included in healthy diets [14–16], like the Mediterranean diet [12] and is a rich source of several nutrients associated with good overall health [14, 17]. Two reviews suggested that fish, and nutrients through which fish is an important dietary source, prevented physical frailty and its individual characteristics [18, 19]. Fish is typically classified based on fat content (fatty vs lean) or the colour of the meat (red vs white). Both methods cover all fish types as white fish can be both fatty (halibut) and lean (cod), and vice versa. As the nutrient composition of lean and fatty fish differs, a healthy diet should include both [20].

Findings from longitudinal, cross-sectional and intervention studies indicate that intake of fish is associated with beneficial health effects in older adults, including healthier ageing [21], reduced risk of frailty [22–24], increased grip strength [25] and improved muscle mass and function [26]. However, results are inconsistent, and no study has specifically investigated the association between different patterns of habitual fish intake and later health outcomes.

The Norwegian dietary guidelines recommend eating fish for dinner two to three times a week and to choose fish as a spread or topping on bread [20]. With its long coastal area and longstanding fishing tradition, fish intake in Norway has traditionally been high compared with other countries [27, 28]. This is especially true for Northern Norway, where fishing has been, and still is, an important part of everyday life [28–30]. Therefore, older individuals from Northern Norway provide a suitable cohort for studying the relationship between fish intake and health-related outcomes.

There are few longitudinal studies on fish intake and pre-frailty [22, 23]. We hypothesize that a frequent fish intake is associated with lower risk of pre-frailty, and that maintaining a high frequency of intake over time reflects some consistency in healthy eating habits which will consequently reduce the risk of pre-frailty. Therefore, building on our previous research on nutrition and pre-frailty/frailty [31], we aimed to elucidate the longitudinal association between fish intake and pre-frailty in an older northern Norwegian, population-based cohort. First, we investigated the association between frequency of intake of lean, fatty and total fish and pre-frailty 8 years later – a follow-up period that we considered to be clinically relevant in terms of a possible implementation of preventive measures. Second, to assess the influence of long-term consistent fish intakes, we investigated the association between consistent low, medium, and high frequency of total fish intake over 21 years and pre-frailty.

## Methods

### The Tromsø Study

The Tromsø Study, described in detail elsewhere [32, 33] is a large population-based study consisting of seven surveys (Tromsø1 to Tromsø7) conducted between 1974 and 2016. Based on the official population registry, total birth cohorts and random samples of residents of the municipality of Tromsø in Northern Norway were invited. In total, 45 473 men and women have participated in one or more surveys [33]. Invitations were sent by mail together with a short questionnaire. On attendance (visit 1), the participants received more comprehensive questionnaires and underwent biological sampling and clinical examinations. A subsample (predefined before study start, but only invited if the person attended visit 1) attended additional clinical examinations (visit 2).

#### Study population

We used data from Tromsø4 (1994–1995), Tromsø6 (2007–2008, baseline survey for main analysis) and Tromsø7 (2015–2016, follow-up survey). Tromsø4 included 27 158 participants (attendance 77%), aged 25–97 [34]. Owing to age-specific questionnaires in Tromsø4, only data from participants aged < 70 years were used in the present study [34]. Tromsø6 included 12 977 participants (66% attendance), aged 30–87 [35]. Tromsø7 included 21 083 participants (65% attendance), aged 40–99 [33].

**Fig. 1:**
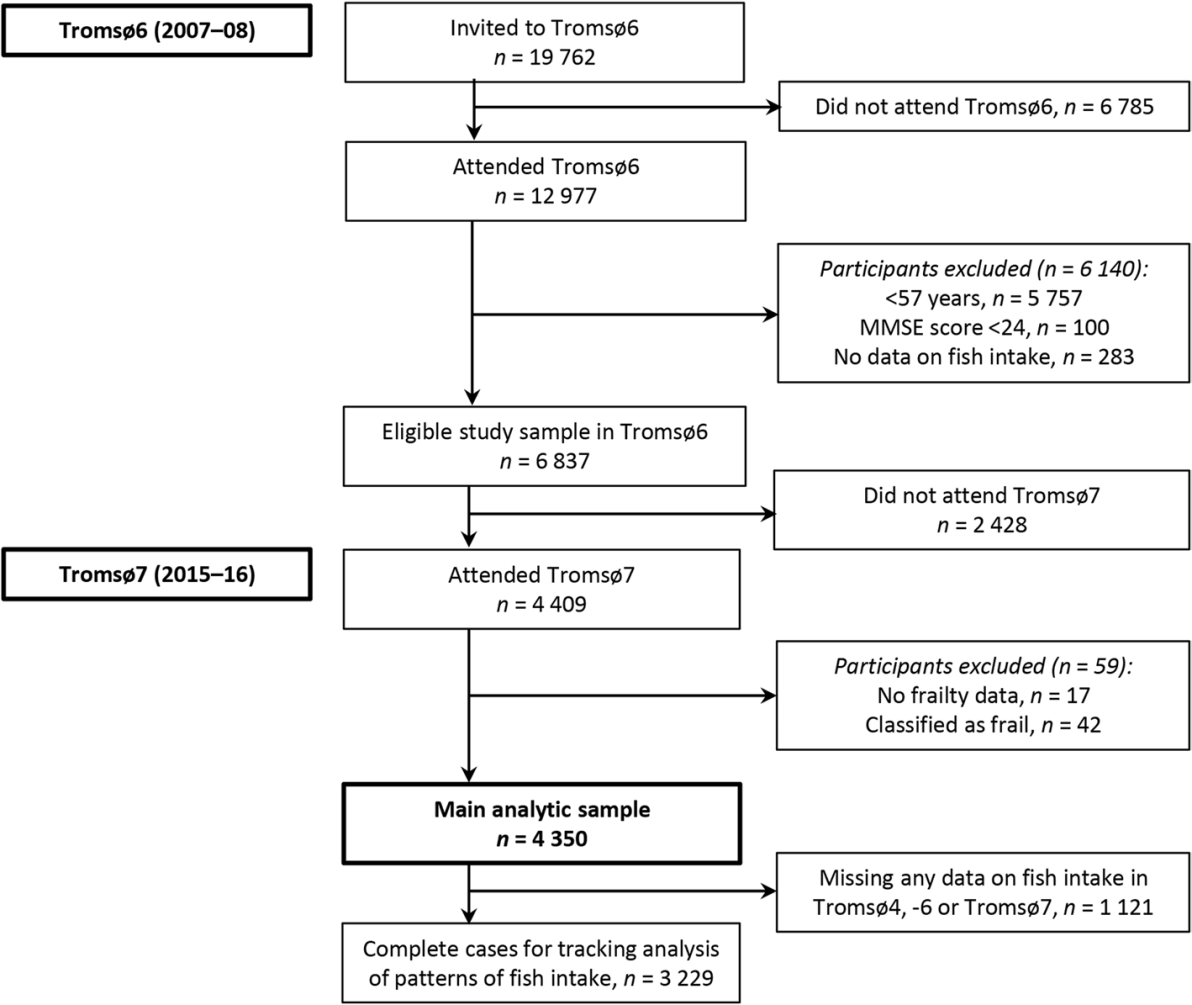
Flow chart of the study population

For the main analysis, baseline was set to Tromsø6 with 8-year follow-up at Tromsø7 (Fig. 1). To ensure an eligible and reliable study sample of appropriate age at follow-up (≥ 65 years), we excluded those younger than 57 years at baseline, those with a Mini-Mental State Examination (MMSE) score < 24, and those with no data on baseline frequency of fish intake. Of the 6837 eligible participants, 4409 also participated at follow-up. At follow-up, we excluded those without any frailty data (*n* = 17) and - given the low prevalence - those classified as frail (*n* = 42), leaving 4350 participants for the main analysis. Among these, a subsample of 3229 participants with complete data on fish intake in all three surveys (Tromsø4, Tromsø6 and Tromsø7) was identified for tracking analysis of patterns of fish intake over 21 years (Fig. 1). For clarity, we will refer to the subsamples as ‘main sample’ (*n* = 4350) and ‘tracking sample’ (*n* = 3229) to distinguish between the two.

### Dietary assessment

Fish intake in all surveys was based on two questions about frequency of intake of lean (e.g., cod, saithe) and fatty (e.g., salmon, trout, mackerel, herring, halibut) fish with answer alternatives ranging from ‘0–1 times a month’ to ‘1–2 times a day’ [36–38] (Table S1). The exact wording of the questions and answers differed slightly across the surveys. To ensure a sufficient number of participants and thus statistical power to perform analyses on the different frequencies of fish intake, the lowest frequency category was merged with the second lowest (‘0–1 times a month’ plus ‘2–3 times a month’), and the highest frequency category was merged with the second highest (‘4–6 times a week’ plus ‘1–2 times a day’). This resulted in three levels of fish intake: ‘0–3 times a month’ (low), ‘1–3 times a week’ (medium) and ‘≥4 times a week’ (high) (Table S1). Total fish intake was estimated by combining frequencies of lean and fatty fish intake. Each frequency interval of lean and fatty fish intake was quantified as total weekly frequency of fish intake (x/week), summed together, and then transformed back into the original frequency intervals (‘categories’) of fish intake.

For assessment of total fish intake over time, stable (low, medium, high) or inconsistent patterns were identified (Table 2). Stable patterns were identified as the same reported frequency of intake in all three surveys (e.g., low, low, low), or two similar frequencies of intake plus one frequency of intake differing by one level. For example, the combination ‘low’, ‘medium’, ‘low’ frequency of intake was also considered a stable low pattern. The remaining patterns were intakes that spread across the three levels of frequency of intake (e.g., low, high, low), and were classified as inconsistent patterns.

### Frailty assessment

In Tromsø6 and at follow-up, a modified versions of Fried’s physical frailty phenotype (Table S2) was used to categorize participants as frail, pre-frail, or robust. Frailty in Tromsø4 was not defined as data were insufficient.

At follow-up, weight loss was defined as answer ‘yes’ to the question: ‘Have you involuntarily lost weight during the last 6 months?’. Low physical activity was defined as the lowest category (‘Mainly reading, watching TV/screen or other sedentary activity’) in the Saltin–Grimby questionnaire [39]. Exhaustion was defined as either of the two highest categories (‘Pretty much’ or ‘Very much’) to the question ‘Have you felt that everything is a struggle during the last week?’, from the Hopkins Symptoms Checklist 10 [40]. Low grip strength and slow walking speed were measured at visit 2 and defined using sex-specific cut-offs, further stratified by body mass index (BMI) quartiles and medium height, respectively, as originally proposed by Fried et al. [5]. BMI was calculated as body weight (kg) divided by height (m) squared (kg/m^2^). Grip strength (kg) was measured using an electric Jamar (PLUS +) dynamometer [33]. The strongest of six measurements was recorded according to the Southampton protocol [41]. Walking speed was assessed by the Short Physical Performance Battery test [42] where participants walked 4 m at their average speed twice. The fastest test was recalculated to seconds per 15 feet to match Fried’s original definition [5].

Frailty was defined in the same way in Tromsø6, except without the walking speed characteristic owing to lack of information. Additionally, grip strength in bar was measured using a Martin-Vigorimeter. Values in bar were calculated to kilopascal before converted to kg using sex-specific conversion factors (women: 2.43, men: 1.68), as according to Neumann et al. [43] to fit Fried's cut-offs [5]. All characteristics were dichotomised. Participants with none of these characteristics were classified as robust, participants with one or two present were classified as pre-frail, and those with three or more characteristics were classified as frail.

### Covariates

Covariates were selected based on empirical knowledge on relevant confounders between diet and pre-frailty. In Tromsø4, body weight (kg) and height (cm) were measure with light clothing and no shoes on an electronic scale. Married/cohabitation included self-reported marriage/partnership/living with spouse/partner. Social support was defined as a yes to the question ‘Do you feel like you have enough good friends?’. Good self-rated health was defined as the two highest (‘Good’ and ‘Very good’) out of five categories to the question ‘What is your current state of health?’. Self-reported smoking status was never, former or daily smoker. Self-reported education level was grouped into primary/lower secondary school (≤ 10 years), upper secondary school and higher education (college/university). Self-reported physical activity level was defined as low if <3 h per week of ‘Light exercise without sweating/being out of breath’. High alcohol intake was defined as an estimated daily intake of ≥ 10 g for women and ≥ 20 g for men, as the Norwegian Directorate of Health advises against intakes above this [44]. Daily alcohol intake was estimated based on self-reported frequency and average units of alcohol consumed. Comorbidity was defined by two or more of the major non-communicable diseases (previous and/or current): cardiovascular disease (angina pectoris, myocardial infarction, stroke), chronic respiratory diseases (chronic bronchitis, asthma), diabetes and cancer. All diseases were self-reported, except cancer, which was obtained from the Norwegian Cancer Registry.

These characteristics were collected in the same way in Tromsø6, with some exceptions; self-reported low physical activity level was defined as the lowest category in the already mentioned Saltin–Grimby questionnaire [39]; alcohol intake was calculated based on the self-reported frequency and average units of alcohol consumed using the first two questions in the Alcohol Use Disorder Identification Test [45]. At visit 2, cognitive function was assessed via the MMSE using a cut-off for normal cognitive function at score 24, which is validated and commonly used for community-dwelling older adults [46].

### Statistical analysis

Characteristics and frequencies of fish intake at different time points are presented as means and counts for the total sample and stratified by follow-up frailty status (Tables 1 and 2). Differences between robust and pre-frail groups were tested using the chi-square test for categorical variables, Student’s *t*-test for continuous variables and Cochran-Armitage test for trend across frequencies of fish intake. Continuous variables were graphically inspected for normality.

**Table 1:** Baseline characteristics and fish intake of main study sample (*n* = 4350)

**Baseline characteristics in Tromsø6**		**Frailty status at follow-up**		
	**All (*n* = 4350)**	**Robust (*n* = 3126)**	**Pre-frail (*n* = 1224)**	***P***^***a***^
Women (%)	51.5	50.3	54.5	0.01
Age (years), mean (SD)	65.1 (5.7)	64.5 (5.5)	66.3 (6.1)	< 0.001
BMI (kg/m^2^), mean (SD)	27.2 (4.1)	26.9 (3.8)	28.1 (4.6)	< 0.001
Cohabitant (%)	76.6	77.7	73.6	0.004
Good social support^b^ (%)	90.0	91.3	86.5	< 0.001
Good self-rated health (%)	66.4	71.7	52.9	< 0.001
Daily smoking (%)				
Never	35.1	36.6	31.7	< 0.001
Previously	50.1	50.4	49.4	
Currently	14.7	13.1	18.9	
Education^c^ (%)				
Lower secondary	33.2	30.5	40.1	< 0.001
Upper secondary	32.4	51.2	48.8	
Higher education	32.5	18.3	11.1	
Sedentary lifestyle (%)	16.1	10.4	31.0	< 0.001
High alcohol intake^d^ (%)	6.4	6.9	5.0	< 0.001
Comorbidity^e^ (%)	4.8	3.7	7.5	< 0.001
MMSE score, mean (SD)	28.3 (1.4)	28.3 (1.4)	28.1 (1.4)	0.02
Cod liver/fish oil supplements (%)	75.9	77.0	73.2	0.008
**Frequency of fish intake**				
Lean fish (%)				
0–3/month	17.1	16.4	18.8	0.1
1–3/week	67.2	67.5	66.6	
≥ 4/week	15.7	16.2	14.6	
Fatty fish (%)				
0–3/month	48.2	46.2	53.6	< 0.001
1–3/week	43.6	45.2	39.6	
≥ 4/week	8.1	8.7	6.8	
Total fish^f^ (%)				
0–3/month	11.1	10.1	13.6	< 0.001
1–3/week	37.3	36.3	39.8	
≥ 4/week	51.7	53.6	46.6	

BMI, body mass index; MMSE, Mini-Mental State Examination; SD, standard deviation.* N* deviates slightly owing to missing data in specific covariates

^a^P-value: Student’s t-test for continuous variables, chi-square test for categorical variables between robust and pre-frail groups

^b^Self-reported satisfactory level of good friends

^c^Primary/secondary school, modern secondary school; technical school, vocational school, 1–2 years senior high school or high school diploma; college/university

^d^Daily alcohol intake ≥10 g (women) or ≥20 g (men)

^e^The presence of ≥2 of the following diseases: cardiovascular disease (angina, heart attack, stroke), pulmonary disease (chronic bronchitis, asthma), diabetes and cancer

^f^The sum of fatty and lean fish intake

**Table 2:** Frequency of fish intake and patterns of total fish intake for tracking sample (*n* = 3229)^a^

**Frequency of fish intake**	**Study waves of the Tromsø Study**			
	**Tromsø4 (1994-1995) **	**Tromsø6 (2007-08)**	**Tromsø7 (2015-16)**	
Lean fish (%)				
0–3/month	12.5	16.8	11.6	
1–3/week	84.9	67.9	74.5	
≥ 4/week	2.5	15.3	13.8	
Fatty fish (%)				
0–3/month	55.4	47.9	44.6	
1–3/week	44.3	44.4	50.0	
≥ 4/week	0.3	7.7	5.5	
Total fish^a^ (%)				
0–3/month	10.0	10.7	7.3	
1–3/week	65.9	37.0	35.2	
≥ 4/week	24.0	52.4	57.5	
**Patterns of fish intake across Tromsø4, Tromsø6, Tromsø7**				
	All (*n* = 3229)	Robust (*n* = 2351)	Pre-frail (*n* = 878)	***P***^***b***^
Stable patterns^c^				< 0.001
Low	4.5	3.7	6.6	
Medium	42.3	41.9	43.4	
High	42.3	44.1	37.7	
Inconsistent^d^	10.9	10.4	12.3	

^a^The sum of fatty and lean fish intake

^b^*P* value: chi-square test

^c^Stable patterns of fish intake defined as the same reported frequency of intake in all three surveys, or two similar frequencies of intake plus one frequency of intake differing by one level

^d^Inconsistent patterns defined as patterns of fish intake that spread across the three levels of frequency of intake

The longitudinal association between frequency of fish intake and pre-frailty was analysed via multivariable logistic regression in two ways: first, the association between frequency of intake of lean, fatty and total fish in Tromsø6 and pre-frailty 8 years later (Table 3). Three multivariable logistic regression models were run, adjusted for relevant Tromsø6 confounders. Model 1 was adjusted for age and sex. Model 2 was additionally adjusted for BMI, education, smoking, physical activity, self-reported health and comorbidity. In addition, to highlight the possible impact of dietary supplement use, model 3 was further adjusted for use of cod liver oil and long-chain omega-3 fatty acids (LCn-3FA) supplements.

**Table 3:** Odds ratios (ORs) and 95% confidence intervals (CIs) for baseline fish intake and 8-year follow-up pre-frailty (*n* = 4350)^a^

**Dietary exposure (Tromsø6)**	**Model 1**		**Model 2**		**Model 3**		***P***_***t*rend**_^b^
	**OR**	**95% CI**	**OR**	**95% CI**	**OR**	**95% CI**	
**Frequency of fish intake**							
Lean fish	(*n* = 4270)		(*n* = 3037)		(*n* = 3037)		
0–3/month	Ref		Ref		Ref		< 0.001
1–3/week	0.82	0.69, 0.98	0.82	0.66, 1.03	0.82	0.66, 1.03	
≥ 4/week	0.69	0.55, 0.88	0.72	0.53, 0.97	0.72	0.53, 0.97	
Fatty fish	(*n* = 4275)		(*n* = 3043)		(*n* = 3043)		
0–3/month	Ref		Ref		Ref		0.04
1–3/week	0.75	0.65, 0.87	0.81	0.68, 0.97	0.81	0.68, 0.97	
≥ 4/week	0.65	0.49, 0.85	0.63	0.44, 0.92	0.63	0.44, 0.92	
Total fish^c^	(*n* = 4195)		(*n* = 3000)		(*n* = 3000)		
0–3/month	Ref		Ref		Ref		< 0.001
1–3/week	0.78	0.62, 0.97	0.87	0.66, 1.15	0.87	0.66, 1.16	
≥ 4/week	0.60	0.48, 0.75	0.68	0.52, 0.90	0.69	0.52, 0.91	

^a^Main analytic sample. *N* deviates owing to missing data in specific adjustment variables

^b^*P* value: Cochran-Armitage test for trend across groups

^c^The sum of fatty and lean fish intake

Model 1: adjusted for Tromsø6 age and sex. Model 2: additionally adjusted for Tromsø6 body mass index, education, comorbidity, smoking, activity level and self-reported health. Model 3: additionally adjusted for Tromsø6 cod liver oil and/or long-chain omega-3-fatty acids supplement use

Second, to elucidate the influence of long-term habitual fish intake, the models were run on the association between different patterns of stability of total fish intake over 21 years (Tromsø4, Tromsø6 and at follow-up) and pre-frailty at follow-up (Table 4). Participants included in the tracking analysis had data on lean and fatty fish intake from all three surveys. A stable low fish intake was chosen as the reference category.

**Table 4:** Odds ratios (ORs) and 95% confidence intervals (CIs) for patterns of fish intake and pre-frailty (*n* = 3229)^a^

Patterns of total fish intake across Tromsø4, Tromsø6, Tromsø7	Model 1 (*n* = 3229)		Model 2 (*n* = 2329)		Model 3 (*n* = 2329)	
	**OR**	**95% CI**	**OR**	**95% CI**	**OR**	**95% CI**
Stable patterns^b^						
Low^c^	Ref		Ref		Ref	
Medium	0.52	0.36, 0.75	0.69	0.44, 1.07	0.69	0.44, 1.07
High	0.41	0.28, 0.59	0.59	0.38, 0.92	0.59	0.38, 0.91
Inconsistent pattern^d^	0.61	0.40, 0.91	0.95	0.57, 1.56	0.94	0.57, 1.56

^a^Tracking sample: complete cases. Participants with available data on all questions on frequency of lean and fatty fish intake in Tromsø4, -6 and -7. *N* deviates owing to missing data in specific adjustment variables

^b^Stable patterns of fish intake defined as the same reported frequency of intake in all three surveys, or two similar frequencies of intake plus one frequency of intake differing by one leve

^c^Reference category

^d^Inconsistent patterns defined as patterns of fish intake that spread across the three levels of frequency of intake

Model 1: adjusted for Tromsø6 age and sex. Model 2: additionally adjusted for Tromsø6 body mass index, education, comorbidity, smoking, activity level and self-reported health. Model 3: additionally adjusted for Tromsø6 cod liver oil and/or long-chain omega-3-fatty acid supplement use

To account for potential influence of already present frailty in the study sample, we repeated the main analysis as a sensitivity analysis in a sample where participants with frailty in Tromsø6 were excluded (Table S4). Further, supplementary analyses were performed to address bias from selective attrition of participants after Tromsø6. First, we compared characteristics of non-attenders after Tromsø6 versus participants who attended follow-up (Table S5). Second, inverse probability of participation weighting (IPPW) [47, 48] was applied to repeat the main analyses in a hypothetical study sample with 100% re-attendance at follow-up (Table S6). This pseudo-population was created through up-weighting characteristics likely to be lost with attrition. Specifically, follow-up participants were weighted by the inverse of their probability of participating at follow-up, to account for the absent weights of the non-attenders. Weights were based on the predicted likelihood of follow-up participation, predicted by the adjustment variables included in model 2, following Metten et al. [47]. Furthermore, we compared the characteristics of participants with complete versus incomplete data on fish intake in the three surveys (Table S7). As a sensitivity analysis to account for missing data, we repeated the tracking analysis in a sample with multiple imputed (MI) data on fish intake in the three surveys (Table S8). Fifty duplicate datasets were created via the predictive mean matching imputation method and estimates were combined with Rubin’s rule [49].

Adjustment variables included in the statistical models were initially chosen from univariate analyses (*P* < 0.2), in addition to clinical importance and considerations about confounding (as was the case for sex and dietary supplements). Subsequently, the multivariable models were built through careful evaluation of the contribution of each variable and comparisons between unrestricted and restricted versions of the model until it had an optimal fit [50]. Age and BMI were included as continuous variables whereas all others were categorical. Owing to the identification of non-linearity, BMI was included in both its linear and its squared form. There were no indications of multicollinearity between the adjustment variables and no statistically significant, clinically plausible interactions. All analyses were performed in STATA/MP 16. *P* values < 0.05 were considered to be statistically significant.

## Results

### Participants’ characteristics and fish intake

In total, 28% (*n* = 1124) of the main study population were classified as pre-frail at follow-up (Table 1). Of these, 84% (*n* = 1031) presented with only one frailty characteristic (Table S9). The most prominent characteristic of physical frailty at follow-up was by far self-reported low physical activity level, which was the only frailty characteristic present in 51% of the pre-frail participants (Table S9). About one third of the participants had missing frailty data, and 23% had missing data on two characteristics. The prevalence of pre-frailty increased with age (Table S10).

In Tromsø6, the mean age was 65 years (range 57–87 years) and 52% were women (Table 1). Pre-frail participants differed from robust participants as they were more likely to be women, older, daily smokers, inactive, lower educated and have higher BMI than robust participants. They were also less likely to be satisfied with self-perceived support from friends and their own health. More pre-frail participants than robust participants lived alone, and the proportion of pre-frail participants with comorbidity was twice as high as among robust participants (Table 1). Three-quarters of all participants used cod liver oil and/or LCn-3FA supplements, more commonly used by robust than by pre-frail participants.

Comparing non-attenders after Tromsø6 (36%) versus participants who re-attended Tromsø7 showed that the latter had notably more favourable health and socioeconomic characteristics but that fish intakes were similar (Table S5).

For the tracking subsample, differences were similar between pre-frail and robust participants as in the main sample (Table S3). Comparing participants with complete versus incomplete data on fish intake in the three surveys showed that complete cases had a slightly more favourable health and socioeconomic profile (Table S7).

In Tromsø6, the main sample ate lean fish more frequently than fatty fish (Table 1). Robust participants ate fatty and total (but not lean) fish more frequently than pre-frail participants. Of the robust participants, 54% had a medium or high intake (≥ 1/week) of fatty fish compared with 46% of pre-frail participants (*P* < 0.001). For total fish, 90% of robust and 86% of pre-frail participants had a medium or high intake (*P* < 0.001).

Also for the tracking sample, lean fish was eaten more frequently than fatty fish at all times (Table 2). The frequency of intake of fatty and total fish appeared to increase between surveys. For fish intake over 21 years, the vast majority had either a stable medium (42%) or stable high (42%) pattern of fish intake (Table 2). A stable low pattern of fish intake was slightly more common among pre-frail than robust participants (7% vs 4%), while a stable high pattern over time was more common among robust than pre-frail participants (44% vs 38%) (*P* < 0.001).

### Fish intake in Tromsø6 and pre-frailty 8 years later

Overall, the main analysis showed that a more frequent fish intake in Tromsø6 was associated with lower odds of pre-frailty 8 years later (*P* value for trend < 0.05) (Table 3). The observed associations from the multivariable model (model 2) and after further adjustment for dietary supplement use (model 3) were similar.

Fully adjusted analysis (model 3) showed that a high intake (≥ 4/week) of lean fish was associated with 28% (OR = 0.72, 95% CI = 0.53, 0.97) lower odds of pre-frailty at follow-up 8 years later compared with a low intake (0–3/month). For fatty fish, a medium (1–3/week) or high intake in Tromsø6 was associated with 19% (OR = 0.81, 95% CI = 0.68, 0.97) and 37% (OR = 0.63, 95% CI = 0.44, 0.92) lower odds of pre-frailty after 8 years, respectively, compared with a low intake. Fully adjusted analysis of total fish intake showed that the odds of pre-frailty after 8 years was 31% lower for participants with a high compared with a low frequency of intake (OR = 0.69, 95% CI = 0.52, 0.91). Results were similar, albeit slightly amplified, in sensitivity analysis excluding pre-frail and frail individual at baseline (Table S4). Fully adjusted sensitivity analyses with IPPW showed no significant association between frequency of fish intake in Tromsø6 and pre-frailty 8 years later (Table S6).

### Patterns of fish intake over 21 years and pre-frailty

Fully adjusted tracking analysis showed that a stable high frequency of intake across Tromsø4, Tromsø6 and Tromsø7 was associated with 41% lower odds of pre-frailty (OR = 0.59, 95% CI = 0.38, 0.91) in Tromsø7, compared with a stable low pattern (Table 4). Results were similar with MI (56% missing data on fish intake) (Table S8).

## Discussion

In the present prospective cohort study, we found that a higher frequency of (lean, fatty and total) fish intake was significantly associated with lower odds of physical pre-frailty after 8 years in older community-dwelling adults in Norway. Moreover, a pattern of consistent high frequency of total fish intake over 21 years was associated with lower odds of pre-frailty.

Overall, the main study population was a relatively healthy sample of older residents in Tromsø, Northern Norway. Considering that individuals with low cognitive skills in Tromsø6 were excluded, alongside the need for physical attendance in the Tromsø study, we assume that the study population is mainly community-dwelling.

The observed prevalence of pre-frailty in the present study was lower than reported among community-dwelling older adults worldwide [51], in Europe [52], and Tromsø5 study participants aged ≥ 70 years in 2001 [53]. These discrepancies may be partly explained by the use of different modifications of Fried’s frailty definition [54]. Moreover, another study from the Tromsø Study has shown increased grip strength in more recent birth cohorts of older participants [55]. Considering that there were 15 years between the measures of frailty status, this may partly explain the differences in frailty prevalence reported in the present study versus the study by Langholz et al. [53]. In line with previous research, the prevalence of pre-frailty in Tromsø7 was higher in women and increased with age [5, 51–53].

The overall relatively high frequency of fish intake observed in all three surveys was somewhat expected, considering that older Norwegians have been found to eat more fish than younger generations and that fish intake, in general, is high in Northern Norway [27–30]. The observed higher frequency of fish intake in the robust compared with the pre-frail participants, taken together with their better health and socioeconomic characteristics, is supported by a recent, large systematic review that found that seafood consumers were more likely to be older, more affluent, educated and physically active and less likely to be smokers compared with non-seafood consumers [56]. In contrast to this, the frequency of fish intake was similar for dropouts after Tromsø6 compared with those re-attending Tromsø7, even though the sociodemographic characteristics in the latter group were slightly more favourable.

### Longitudinal associations between frequency of fish intake and pre-frailty

Our findings suggest that how often one eats fish in late adulthood may influence later odds of pre-frailty. This emphasizes the importance for this age group of adhering to the Norwegian Dietary Guidelines’ recommendations of eating fish two to three times a week [20]. A benefit and risk assessment of fish in the Norwegian diet recently concluded that there were positive health benefits associated with increasing the Norwegian adult’s fish intake to the upper end of the recommended intake range [57]. Although not directly comparable, our results agree with this. The strengths of the observed associations between frequency of fish intake and pre-frailty increased with higher frequency of intake.

As the existing literature on fish intake and pre-frailty is particularly scarce, the comparison of our results is limited to studies focusing on frailty or frailty-related outcomes.

The observed beneficial association between increased frequency of fatty fish intake and later pre-frailty is supported by findings from a longitudinal Spanish study in 1592 community-dwelling adults aged ≥60 years conducted by García-Esquinas et al. [21]. They observed an inverse association between increased daily estimated intake of fatty fish and accumulation of age-related health deficits 6 years later. The health deficit accumulation index is another widespread and more comprehensive measure of frailty than Fried's physical phenotype [58]. In addition, a cross-sectional study conducted in rural coastal Ecuador showed a stepwise decrease in frailty scores for each additional weekly serving of fatty fish consumed among community dwellers aged 60–69 years [23]. Notably, there was no association between fish intake and frailty status in the participants aged ≥70 years, for whom the authors speculated that the effects of age superseded the positive effects of fatty fish.

For lean fish, the observed beneficial association between high intakes and pre-frailty is in accordance with a Saudi Arabian intervention study which showed that eating lean fish for lunch twice a week for 10 weeks significantly increased muscle mass and walking speed in 22 adults (≥50 years) [26]. However, in the longitudinal study by García-Esquinas et al., they did not find any association between intake of lean fish and healthy ageing [21].

In line with our findings, García-Esquinas et al. did, however, observe reduced deficit accumulation scores with increasing quintiles of total fish intake [21]. Furthermore, an Irish cross-sectional study in community-dwelling older adults (≥ 65 years) observed significantly higher odds of Fried’s physical frailty among those in the lowest tertile of intake of fish and fish products compared with the highest [22]. In addition, a cross-sectional study in Japanese female outpatients with rheumatoid arthritis found that, of 20 foods assessed, fish intake more than twice a week was identified as independently negatively associated with pre-frailty/frailty (pre-frail and frailty combined as outcome) [24].

Taken together, the comparability of the results from these studies with our study is somewhat limited. The levels of fish intake differs, and all, except the study by O’Connell et al. [22], use different frailty definitions, have no mention of dietary supplements, and include study populations and settings that differ greatly from the relatively healthy community-dwelling older adults from Northern Norway [21–24, 26].

Our results from the tracking analysis showing lower odds of pre-frailty from a consistent high frequency of intake compared with consistent low frequency of intake was as hypothesized. To the best of our knowledge, no earlier study has tracked fish intake over time in relation to frailty or other age-related health outcomes.

Some of the plausible biological pathways between nutrients in fish and health that could be relevant in the observed association between fish intake and pre-frailty include vitamin D’s beneficial effect on bone health and muscle function [14, 19, 59]; the anti-inflammatory properties of LCn-3FA [14, 59, 60], or lower rate of muscle loss from increased intake of high-quality fish protein [14, 59, 61]. However, it is important to emphasize that owing to the nature of the frequency data and the long follow-up times, what we have truly assessed is the *habit* of eating fish and not the biological properties of the fish and its nutrients. Moreover, one could speculate that the observed protective effect of frequent fish intake, in participants where fish makes up a large proportion of their total diet, simply reflects a subsequent lower intake of other and perhaps less healthy foods.

### Strengths and limitations

A limitation of the study is the self-reported data, which introduces risk of information bias. Unfortunately, self-reported dietary data are typically misreported, either consciously or unconsciously [62]. Given the general status of fish as a healthy food [63], one could speculate that fish intakes are over-reported. Another limitation is that the two variables on fish intake that provided the basis for the analyses were too crude to capture the participant’s absolute intake. Moreover, the variables depend on the participants' prior knowledge on what constitutes fatty and lean fish and this may have introduced uncertainty to the study. Additional information about intake of other fish products and fish spread was available in the different surveys, albeit at different levels, and, therefore, to facilitate comparability between time points, the focus was kept on the two variables lean and fatty fish.

Another limitation is the variation within the stable patterns of fish intake, owing to the definition criteria which allows for one differing frequency of intake. Thus, patterns might vary substantially within categories, depending on whether the 'one off' is a higher or lower frequency than the other two, or in what survey the different frequency of intake was reported.

Selection bias is a common limitation in cohort studies, because participants tend to be healthier and have better socioeconomic status than non-attenders [64]. This is emphasized by the overall good health of the study population and the low prevalence of pre-frailty in Tromsø7. In addition, the predominance of pre-frail participants with a frailty score of only 1, where many had low physical activity level as their only frailty characteristic may reflect that the pre-frail group largely consisted of sedentary, but otherwise healthy, individuals. The slightly weaker association observed between frequency of fish intake and pre-frailty in the IPPW sensitivity analysis could be explained by a lower degree of selection bias. Considering the observed differences between those who participated in Tromsø7 versus the non-attenders, the pseudo-population included in the IPPW analysis, with 100% participation in Tromsø7, was older and more heterogeneous than the main study population. Thus, the effects of age and poorer health might to some extent have superseded the positive effects of frequent fish intake on later pre-frailty in these participants. Notably, the substantial level of missing frailty data might have contributed to an incorrectly measured prevalence of pre-frailty and biased results.

With these limitations in mind, the study’s results should be interpreted somewhat cautiously and their generalization is limited to relatively healthy, community-dwelling, older Norwegian adults. However, in favour of our findings of an inverse association between increased frequency of fish intake and pre-frailty after 8 years, were the results from the sensitivity analysis performed after exclusion of baseline pre-frail/frail participants and the tracking analysis with MI.

The strengths of the study include its longitudinal study design, the large study sample, and the use of validated instruments for frailty assessment. In addition, the available data were scrutinized to thoroughly assess the fish–pre-frailty association by investigating lean, fatty, and total fish, the impact of different lengths of follow-up and the specific adjustment for use of cod liver oil and LCn-3FA supplements. Furthermore, the performance of supplementary analyses to account for inherent and unavoidable weaknesses of observational studies, like the already mentioned risk of attrition and the influence of missing data, adds transparency and value to the interpretation of the results.

## Conclusions

This study shows that higher frequency of fish intake among middle-aged and older community-dwelling adults reduce later odds of pre-frailty. Thus, our study emphasizes the importance of a frequent fish intake to prevent pre-frailty and facilitate healthy ageing.

## Supplementary Information

**Additional file 1:** **Table S1.** Original and modified categories of frequency of fish intake in the Tromsø Study. **Table S2.** Modifications of the frailtyphenotype in the Tromsø7 Study (2015–2016). **Table S3.** Characteristics of tracking sample in Tromsø4 and Tromsø6 (*n*=3229). **Table S4.** Odds ratios (ORs) and 95% confidence intervals (CIs) for fish intake and 8-year follow-up pre-frailty after exclusion of baseline frailty (*n* = 3219)^a^. **Table S5.** Characteristics of participants in Tromsø6 by Tromsø7 participation status (*n* = 6837)^a^. **Table S6.** Odds ratios (ORs) and 95% confidence intervals (CIs) for baseline fish intake and pre-frailty with inverse probability weights^a^ (*n* = 6183)^b^. **Table S7.** Characteristics of participants in Tromsø6 with complete and incomplete data on fish intake (*n* = 5750)^a^. **Table S8.** Odds ratios (ORs) and 95% confidence intervals (CIs) for patterns of fish intake and pre-frailty using multiple imputation (MI)^a^ (*n* = 5750)^b^. **Table S9.** Onset of physical frailty characteristics in Tromsø7 (*n* = 4350)^a^. **Table S10.** Frailty prevalence in Tromsø7 stratified by age (*n* = 4350)^a^.

## Data Availability

The legal restriction on data availability is set by the Tromsø Study Data and Publication Committee in order to control for data sharing, including publication of datasets with the potential of reverse identification of de-identified sensitive participant information. The data that support the findings of this study are available from the Tromsø Study but restrictions apply to the availability of these data, which were used under license for the current study, and so are not publicly available. Data are however available from the authors upon reasonable request and with permission of the Tromsø Study Data and Publication Committee. Contact information: The Tromsø Study, Department of Community Medicine, Faculty of Health Sciences, UiT The Arctic University of Norway; e-mail: tromsous@uit.no. A detailed overview of the data collection process and links to the main questionnaires, can be found on the Tromsø Study’s website (https://uit.no/research/tromsostudy). All variables collected in the Tromsø Study can be found in NESSTAR (http://tromsoundersokelsen.uit.no/tromso/).

## Abbreviations

BMI: Body mass index
CI: Confidence interval
IPPW: Inverse participation probability weighting
LCn-3FA: Long-chain omega-3 fatty acids
MI: Multiple imputation
MMSE: Mini-Mental State Examination
OR: Odds ratio
