# Supplementary material for: Fish intake and pre-frailty in Norwegian older adults - a prospective cohort study: the Tromsø Study 1994–2016
Source: BMC Geriatr. 2023 Jul 5;23:411. doi: 10.1186/s12877-023-04081-z (PMC10324151; doi:10.1186/s12877-023-04081-z)
Supplement: Supplementary file 1 — Additional file 1: Table S1. Original and modified categories of frequency of fish intake in the Tromsø Study. Table S2. Modifications of the frailtyphenotype in the Tromsø7 Study (2015–2016). Table S3. Characteristics of tracking sample in Tromsø4 and Tromsø6 (n=3229). Table S4. Odds ratios (ORs) and 95% confidence intervals (CIs) for fish intake and 8-year follow-up pre-frailty after exclusion of baseline frailty (n = 3219)a. Table S5. Characteristics of participants in Tromsø6 by Tromsø7 participation status (n = 6837)a. Table S6. Odds ratios (ORs) and 95% confidence intervals (CIs) for baseline fish intake and pre-frailty with inverse probability weightsa (n = 6183)b. Table S7. Characteristics of participants in Tromsø6 with complete and incomplete data on fish intake (n = 5750)a. Table S8. Odds ratios (ORs) and 95% confidence intervals (CIs) for patterns of fish intake and pre-frailty using multiple imputation (MI)a (n = 5750)b. Table S9. Onset of physical frailty characteristics in Tromsø7 (n = 4350)a. Table S10. Frailty prevalence in Tromsø7 stratified by age (n = 4350)a. [file 12877_2023_4081_MOESM1_ESM.docx]

**Additional file 1: Table S1–S10**

**Table S1** Original and modified categories of frequency of fish intake in the Tromsø Study

| **Frequency of fish intake** | **Study waves of the Tromsø Study** | | | | | | | |
| --- | --- | --- | --- | --- | --- | --- | --- | --- |
|  | **Tromsø4 (1994–1995)**^a^ | |  | **Tromsø6 (2007–2008)** | |  | **Tromsø7 (2015–2016)** | |
| **Lean fish** | **How many times per week do you usually eat lean fish (e.g. cod) for dinner?** | |  | **How often do you usually eat lean fish?** | |  | **How often do you usually eat lean fish (cod, saithe)?** | |
|  | *Original* | *Modified* |  | *Original* | *Modified* |  | *Original* | *Modified* |
| ***Low*** | Never | 0–3 times a month |  | 0–1 times a month | 0–3 times a month |  | 0–1 times a month | 0–3 times a month |
|  | <1 |  |  | 2–3 times a month |  |  | 2–3 times a month |  |
| ***Medium*** | 1 | 1–3 times a week |  | 1–3 times a week | 1–3 times a week |  | 1–3 times a week | 1–3 times a week |
|  | 2–3 |  |  |  |  |  |  |  |
| ***High*** | 4–5 | >4 times a week |  | 4–6 times a week | >4 times a week |  | 4–6 times a week | >4 times a week |
|  | Approx. daily |  |  | 1–2 times a day |  |  | ≥1 time a day |  |
| **Fatty fish** | **How many times per week do you usually eat fatty fish (e.g. salmon/redfish) for dinner?** | |  | **How often do you usually eat fatty fish (e.g. salmon, trout, mackerel, herring, halibut, redfish)?** | |  | **How often do you usually eat fatty fish (salmon, trout, redfish, mackerel, herring, halibut)?** | |
|  | *Original* | *Modified* |  | *Original* | *Modified* |  | *Original* | *Modified* |
| ***Low*** | Never | 0–3 times a month |  | 0–2 times a month | 0–3 times a month |  | 0–2 times a month | 0–3 times a month |
|  | <1 |  |  | 2–3 times a month |  |  | 2–3 times a month |  |
| ***Medium*** | 1 | 1–3 times a week |  | 1–3 times a week | 1–3 times a week |  | 1–3 times a week | 1–3 times a week |
|  | 2–3 |  |  |  |  |  |  |  |
| ***High*** | 4–5 | >4 times a week |  | 4–6 times a week | >4 times a week |  | 4–6 times a week | >4 times a week |
|  | Approx. daily |  |  | 1–2 times a day |  |  | ≥1 time a day |  |

Approx.: approximately. ^a^Information collected only among participants in Tromsø4 aged <70 years. Participants aged ≥70 years (*n* = 15) were given a different questionnaire with other, and incomparable, frequency intervals of fatty and lean fish intake.

**Table S2** Modifications of the frailty phenotype in the Tromsø7 Study (2015–2016)

|  | **Criteria for frailty by Fried et al. 2001** | | **Criteria for frailty in Tromsø7** | |
| --- | --- | --- | --- | --- |
| Weight loss | Self-reported, from the question ‘In the last year, have you lost more than 10 pounds unintentionally (i.e. not due to dieting or exercise)?’. If yes, then frail for weight loss criterion. Or, at follow-up: ≥5% unintentional loss of body weight in prior year (by direct measurement of weight at follow-up) | | Self-reported, based on a question from the Malnutrition Universal Screening Tool: ‘Have you involuntarily lost weight during the last 6 months?’. If yes, then frail for the weight loss criterion | |
| Exhaustion | Self-reported, based on two questions from the Center for Epidemiologic Studies Depression Scale:  (a) I felt that everything I did was an effort  (b) I could not get going.  ‘How often in the last week did you feel this way?’  0 = rarely or none of the time (<1 day)  1 = some or a little of the time (1–2 days)  2 = a moderate amount of the time (3–4 days)  3 = most of the time  Answer 2 or 3 to either of these questions led to categorisation as frail for the exhaustion criterion | | Self-reported, based on the Hopkins Symptoms Checklist 10: ‘Have you experienced any of this the last week: That everything is a struggle?’  1 = No complaint  2 = Little complaint  3 = Pretty much  4 = Very much  Answer 3 or 4 led to categorisation as frail by the exhaustion criterion | |
| Physical activity | Self-reported, based on the Minnesota Leisure Time Activity short questionnaire asking about walking, chores (moderately strenuous), mowing the lawn, raking, gardening, hiking, jogging, biking, exercise cycling, dancing, aerobics, bowling, golf, singles tennis, doubles tennis, racquetball, calisthenics, swimming. Kilocalories per week expended were calculated using standardised algorithm. The lowest 20% was identified for each sex  *Frailty cut-off for physical activity:*  *Men*: <383 kcal of physical activity per week  *Women*: <270 kcal of physical activity per week | | Self-reported, based on Saltin–Grimby Physical Activity Level Scale. Describe your exercise and physical exertion in leisure time over the last year:  1 = Reading, watching TV/screen or other sedentary activity?  2 = Walking, cycling or other forms of exercise at least 4 hours a week?  3 = Participation in recreational sports, heavy gardening, snow shovelling etc. at least 4 hours a week?  4 = Participation in hard training or sports competitions, regularly several times a week  Answer 1 led to categorisation as frail for the physical activity criterion | |
| Walking speed | Time to Walk test: walk 15 feet at usual pace (s). Stratified by sex and height (gender-specific cut-off at medium height). Lowest 20% were identified, resulting in the following cut-off for the walking speed criterion of frailty: | | Short Physical Performance Battery walking test: fastest (s) of two times to walk 4 m on average pace. Stratified by sex and height. Calculated from s/4 m to s/15 feet (4.572 m)for adaption to Fried’s criteria: (s/4 m) × 1.143 = s/4.572 m | |
|  | *Men* | *Cut-off (s)* | *Men* | *Cut-off (s)* |
|  | Height ≤173 cm | ≥7 | Height ≤173 cm | ≥7 |
|  | Height >173 cm | ≥6 | Height >173 cm | ≥6 |
|  |  |  |  |  |
|  | *Women* | *Cut-off (s)* | *Women* | *Cut-off (s)* |
|  | Height ≤159 cm | ≥7 | Height ≤159 cm | ≥7 |
|  | Height >159 cm | ≥6 | Height >159 cm | ≥6 |
| Grip strength | Measured by Jamar dynamometer (kg), maximal strength of three trials in dominant hand. Stratified by sex and BMI quartiles. Lowest 20% were identified, resulting in the following cut-off for frailty: | | Measured by Jamar dynamometer (kg), maximal of three trials in each hand (six measurements). Stratified by sex and BMI quartiles: | |
|  | *Men* | *Cut-off (kg)* | *Men* | *Cut-off (kg)* |
|  | BMI ≤24 | ≤29 | BMI ≤24 | ≤29 |
|  | BMI 24.1**–**26 | ≤30 | BMI 24.1**–**26 | ≤30 |
|  | BMI 26.1**–**28 | ≤30 | BMI 26.1**–**28 | ≤30 |
|  | BMI >28 | ≤32 | BMI >28 | ≤32 |
|  |  |  |  |  |
|  | *Women* | *Cut-off (kg)* | *Women* | *Cut-off (kg)* |
|  | BMI ≤23 | ≤17 | BMI ≤23 | ≤17 |
|  | BMI 23.1**–**26 | ≤17.3 | BMI 23.1**–**26 | ≤17.3 |
|  | BMI 26.1**–**29 | ≤18 | BMI 26.1**–**29 | ≤18 |
|  | BMI >29 | ≤21 | BMI >29 | ≤21 |
| **Frailty score** | 0 = Not frail/robust  1**–**2 = Intermediate/pre-frail  ≥3 = Frail | | 0 = Not frail/robust  1**–**2 = Intermediate/pre-frail  ≥3 = Frail | |

**Table S3** Characteristics of tracking sample in Tromsø4 and Tromsø6 (n=3229)

| **Characteristics** | **Study waves of the Tromsø Study** | | | | | | | | |
| --- | --- | --- | --- | --- | --- | --- | --- | --- | --- |
|  | **Tromsø4** | | |  |  | **Tromsø6** | | |  |
|  | **All**  **(*n* = 3229)** | **Robust**  **(*n* = 2351)** | **Pre-frail**  **(*n* = 878)** | ***P^a^*** |  | **All**  **(*n* = 3229)** | **Robust**  **(*n* = 2351)** | **Pre-frail**  **(*n* = 878)** | ***P^a^*** |
| Women (%) | 50.1 | 49.0 | 53.1 | 0.04 |  | 50.1 | 49.0 | 53.1 | 0.04 |
| Age (years), mean (SD) | 51.9 (5.4) | 51.5 (5.2) | 53.1 (5.7) | <0.001 |  | 64.9 (5.4) | 64.5 (5.2) | 66.1 (5.7) | <0.001 |
| BMI (kg/m^2^), mean (SD) | 25.7 (3.5) | 25.4 (3.3) | 26.4 (3.8) | <0.001 |  | 27.2 (4.0) | 26.8 (3.7) | 28.2 (4.6) | <0.001 |
| Cohabitant (%) | 85.4 | 85.9 | 84.2 | 0.2 |  | 79.0 | 79.9 | 76.8 | 0.05 |
| Good social support^b^ (%) | 82.3 | 83.0 | 80.1 | 0.06 |  | 90.5 | 91.6 | 87.5 | 0.001 |
| Good self-rated health (%) | 71.7 | 75.2 | 62.3 | <0.001 |  | 66.5 | 71.5 | 53.4 | <0.001 |
| Daily smoking (%)  Never  Previously  Currently | 34.6  37.1  28.3 | 36.6  37.1  26.3 | 29.2  37.2  33.6 | <0.001 |  | 35.1  52.4  13.6 | 36.8  51.1  12.1 | 30.5  52.1  17.3 | <0.001 |
| Education^c^ (%)  Lower secondary  Upper secondary  Higher education | 32.3  36.1  31.7 | 30.3  35.9  33.8 | 37.6  36.4  26.0 | <0.001 |  | 31.0  35.7  33.3 | 28.9  35.3  35.9 | 36.5  36.9  26.6 | <0.001 |
| Sedentary lifestyle (%) | 32.5 | 29.2 | 41.4 | <0.001 |  | 15.0 | 9.9 | 29.1 | <0.001 |
| High alcohol intake^d^ (%) | 1.9 | 2.0 | 1.8 | 0.7 |  | 6.9 | 7.3 | 5.9 | 0.15 |
| Comorbidity^e^ (%) | 0.9 | 0.9 | 0.9 | 0.9 |  | 4.7 | 3.4 | 8.2 | <0.001 |
| MMSE score |  |  |  |  |  | 28.3 (1.4) | 28.3 (1.4) | 28.1 (1.4) | 0.008 |
| Cod liver/fish oil supplements (%) | 48.2 | 49.2 | 45.6 | 0.1 |  | 76.3 | 77.5 | 72.9 | 0.006 |

BMI, body mass index; MMSE, Mini-Mental State Examination; SD, standard deviation. *N* deviates slightly owing to missing data in specific covariates. ^a^P-value: Student’s t-test for continuous variables, chi-square test for categorical variables between robust and pre-frail groups. ^b^Self-reported satisfactory level of good friends. ^c^Primary/secondary school, modern secondary school; technical school, vocational school, 1-2 years senior high school or high school diploma; college/university. ^d^Daily alcohol intake ≥10 g (women) or ≥20 g (men). ^e^The presence of ≥2 of the following diseases: cardiovascular disease (angina, heart attack, stroke), pulmonary disease (chronic bronchitis, asthma), diabetes and cancer.

**Table S4** Odds ratios (ORs) and 95% confidence intervals (CIs) for fish intake and 8-year follow-up pre-frailty after exclusion of baseline frailty (*n* = 3219)^a^

| **Dietary exposure (Tromsø6)** | **Model 1** | | **Model 2** | | **Model 3** | | ***P_t_*_rend_**^b^ |
| --- | --- | --- | --- | --- | --- | --- | --- |
|  | **OR** | **95% CI** | **OR** | **95% CI** | **OR** | **95% CI** |  |
| **Frequency of fish intake** |  |  |  |  |  |  |  |
| Lean fish | (n = 3158) | | (n = 2379) | | (n = 2379) | |  |
| 0–3/month | Ref. |  | Ref. |  | Ref. |  | 0.3 |
| 1–3/week | 0.87 | 0.69, 1.10 | 0.74 | 0,57, 0.98 | 0.74 | 0.56, 0.98 |  |
| ≥4/week | 0.73 | 0.54, 1.00 | 0.62 | 0.43, 0.89 | 0.61 | 0.43, 0.89 |  |
| Fatty fish | (n = 3172) | | (n = 2399) | | (n = 2399) | |  |
| 0–3/month | Ref. |  | Ref. |  | Ref. |  | <0.001 |
| 1–3/week | 0.74 | 0.62, 0.89 | 0.71 | 0.57, 0.87 | 0.71 | 0.57, 0.88 |  |
| ≥4/week | 0.59 | 0.41, 0.84 | 0.39 | 0.24, 0.64 | 0.40 | 0.24, 0.65 |  |
| Total fish^c^ | (n = 3110) | | (n = 2354) | | (n = 2354) | |  |
| 0–3/month | Ref. |  | Ref. |  | Ref. |  | <0.001 |
| 1–3/week | 0.84 | 0.62, 1.12 | 0.75 | 0.53, 1.06 | 0.75 | 0.54, 1.06 |  |
| ≥4/week | 0.62 | 0.46, 0.82 | 0.52 | 0.37, 0.73 | 0.53 | 0.38, 0.74 |  |

^a^Main analytic sample after exclusion of participants defined as frail or pre-frail in Tromsø6. *N* deviates owing to missing data in specific adjustment variables. ^b^*P* value: Cochran-Armitage test for trend across groups. ^c^The sum of fatty and lean fish intake. **Model 1:** adjusted for Tromsø6 age and sex. **Model 2:** additionally adjusted for Tromsø6 body mass index, education, comorbidity, smoking and self-reported health. **Model 3:** additionally adjusted for Tromsø6 cod liver oil and/or long-chain omega-3-fatty acids supplement use.

**Table S5** Characteristics of participants in Tromsø6 by Tromsø7 participation status (*n* = 6837)^a^

| **Tromsø6 characteristics** | **Did not attend Tromsø7**  **(*n* = 2428)** | **Attended Tromsø7**  **(*n* = 4409)** | ***P^b^*** |
| --- | --- | --- | --- |
| Women (%) | 52.4 | 51.7 | 6 |
| Age (years), mean (SD) | 70.1 (8.0) | 65.1 (5.7) | <0.001 |
| BMI (kg/m^2^), mean (SD) | 27.0 (4.4) | 27.2 (4.1) | 0.05 |
| Cohabitation (%) | 65.1 | 76.4 | <0.001 |
| Good self-rated health (%) | 49.3 | 66.0 | <0.001 |
| Daily smoking (%) | 22.0 | 14.8 | <0.001 |
| Education^c^ (%)  Lower secondary  Upper secondary  Higher education | 46.2  30.3  23.5 | 33.4  34.3  32.3 | <0.001 |
| Sedentary lifestyle (%) | 29.2 | 16.4 | <0.001 |
| High alcohol intake^d^ (%) | 4.9 | 6.3 | 0.02 |
| Comorbidity^e^ (%) | 12.8 | 4.9 | <0.001 |
| MMSE score, mean (SD) | 28.0 (1.5) | 28.3 (1.4) | <0.001 |
| Cod liver/fish oil supplement use (%) | 74.8 | 76.1 | 0.2 |
| **Fish intake** | | | |
| Lean fish  0–3/month  1–3/week  ≥4/week | 20.8  62.0  17.2 | 17.2  67.1  15.7 | <0.001 |
| Fatty fish  0–3/month  1–3/week  ≥4/week | 49.5  40.8  9.7 | 48.3  43.5  8.2 | 0.03 |
| Total fish^f^  0–3/month  1–3/week  ≥4/week | 14.4  35.2  50.5 | 11.2  37.2  51.6 | 0.001 |

BMI, body mass index; MMSE, Mini-Mental State Examination; SD, standard deviation. ^a^Participants in Tromsø6 <57 years, MMSE score >24 with data on lean and/or fatty fish intake. *N* deviates slightly owing to missing data in specific covariates. ^b^*P* value: Student’s t-test for continuous variables, chi-square test for categorical variables. ^c^Primary/secondary school, modern secondary school; technical school, vocational school, 1–2 years senior high school or high school diploma; college/university. ^d^Daily alcohol intake ≥10 g (women) or ≥20 g (men). ^e^The presence of two or more of the following diseases: cardiovascular disease (angina, heart attack, stroke), pulmonary disease (chronic bronchitis, asthma), diabetes and cancer. ^f^The sum of fatty and lean fish intake.

**Table S6** Odds ratios (ORs) and 95% confidence intervals (CIs) for baseline fish intake and pre-frailty with inverse probability weights^a^ (*n* = 6183)^b^

| **Dietary exposure (Tromsø6)** | **Model 1** | | **Model 2** | | **Model 3** | |
| --- | --- | --- | --- | --- | --- | --- |
|  | **OR** | **95% CI** | **OR** | **95% CI** | **OR** | **95% CI** |
| Lean fish  0–3/month  1–3/week  ≥4/week | Ref.  0.79  0.69 | 0.65, 0.96  0.53, 0.91 | Ref.  0.84  0.79 | 0.66, 1.07  0.58, 1.09 | Ref.  0.84  0.79 | 0.66, 1.07  0.57, 1.08 |
| Fatty fish  0–3/month  1–3/week  ≥4/week | Ref.  0.79  0.72 | 0.67, 0.92  0.53, 0.99 | Ref.  0.82  0.71 | 0.68, 0.99  0.49, 1.04 | Ref.  0.83  0.71 | 0.69, 1.00  0.49, 1.04 |
| Total fish^c^  0–3/month  1–3/week  ≥4/week | Ref.  0.79  0.62 | 0.61, 1.01  0.49, 0.79 | Ref.  0.90  0.75 | 0.66, 1.22  0.55,1.01 | Ref.  0.90  0.75 | 0.66, 1.22  0.55, 1.01 |

^a^Probability of re-attending Tromsø7 estimated based on Tromsø6 characteristics age, sex, body mass index, physical activity, comorbidity, education and self-reported health. Subsequently, inverse weights of the estimated probability of attendance were calculated and applied to the study population. This created a pseudopopulation with 100% re-attendance in which characteristics of non-attenders were up-weighted. ^b^Hypothetical study population based on Tromsø6 participants (*n* = 6837). ^c^The sum of fatty and lean fish intake. **Model 1:** adjusted for Tromsø6 age and sex. **Model 2:** additionally adjusted for Tromsø6 BMI, education, comorbidity, smoking, activity level and self-reported health. **Model 3:** additionally adjusted for Tromsø6 cod liver oil and/or long-chain omega-3-fatty acid supplement use.

**Table S7** Characteristics of participants in Tromsø6 with complete and incomplete data on fish intake (*n* = 5750)^a^

| **Characteristics in Tromsø6** | **Incomplete data on fish intake (*n*= 2521)** | **Complete data on fish intake (*n*= 3229)** | ***P***^b^ |
| --- | --- | --- | --- |
| Women (%) | 52.8 | 50.1 | 0.10 |
| Age (years), mean (SD) | 62.9 (6.2) | 5.4 | <0.001 |
| BMI (kg/m^2^), mean (SD) | 27.3 (4.2) | 4.0 | 0.3 |
| Cohabitant (%) | 69.2 | 79.0 | 0.1 |
| Good self-rated health (%) | 65.3 | 66.5 | 0.7 |
| Daily smoking (%) | 18.7 | 13.6 | <0.001 |
| Education^c^ (%)  Lower secondary  Upper secondary  Higher education | 58.2  21.5  20.3 | 31.2  35.6  33.2 | <0.001 |
| Sedentary lifestyle (%) | 19.2 | 15.0 | 0.003 |
| High alcohol intake^d^ (%) | 4.6 | 6.9 | 0.009 |
| Comorbidity^e^ (%) | 4.8 | 4.7 | 0.9 |
| MMSE score | 28.3 (1.4) | 28.3 (1.4) | 0.8 |

BMI, body mass index; MMSE, Mini-Mental State Examination; SD, standard deviation. *N* deviates slightly owing to missing data in specific covariates. ^a^Study sample resembles eligible participants in Tromsø6 (see Figure 1) except for that Tromsø7 attendance was not an exclusion criterion, and the exclusion criterion ‘No data on fish intake in Tromsø6’ was replaced by ‘No data on fish intake in Tromsø4, Tromsø6 or Tromsø7’ (i.e. not at all). Thus, participants are of eligible age and with data on minimum one fish variable from any of the three surveys. ^b^*P* value: Student’s *t*-test for continuous variables, chi-square test for categorical variables. ^c^Primary/secondary school, modern secondary school; technical school, vocational school, 1–2 years senior high school or high school diploma; college/university. ^d^Daily alcohol intake ≥10 g (women) or ≥20 g (men). ^e^The presence of two or more of the following diseases: cardiovascular disease (angina, heart attack, stroke), pulmonary disease (chronic bronchitis, asthma), diabetes and cancer.

**Table S8** Odds ratios (ORs) and 95% confidence intervals (CIs) for patterns of fish intake and pre-frailty using multiple imputation (MI)^a^ (*n* = 5750)^b^

| **Patterns of total fish intake** | **Model 1 (*n* = 5750)** | | **Model 2 (*n* = 5750)** | | **Model 3 (*n* = 5750)** | |
| --- | --- | --- | --- | --- | --- | --- |
|  | **OR** | **95% CI** | **OR** | **95% CI** | **OR** | **95% CI** |
| Stable^c^  Low^d^  Medium  High  Inconsistent^e^ | Ref.  0.64  0.45  0.66 | 0.48, 0.85  0.34, 0.60  0.47, 0.92 | Ref.  0.76  0.57  0.77 | 0.55, 1.03  0.42, 0.77  0.54, 1.12 | Ref.  0.76  0.57  0.78 | 0.55, 1.03  0.42, 0.78  0.54, 1.12 |

^a^Multiple imputation (MI) was performed to address missing data on frequency of intake of fatty and lean fish in Tromsø4, Tromsø6 and Tromsø7. These variables provided the basis for the exposure variable ‘Patterns of total fish intake’ in this analysis. Fifty duplicate datasets were created via predictive mean matching imputation method. The imputation model included the outcome (pre-frailty) and all descriptive variables collected in Tromsø6 (see Table 1). Estimates from the 50 imputed datasets were combined with Rubin’s rules to obtain ORs and 95% CIs. ^b^Study sample resembles eligible participants in Tromsø6 (see Figure 1) except for that Tromsø7 attendance was not an exclusion criterion, and the exclusion criterion ‘No data on fish intake in Tromsø6’ was replaced by ‘No data on fish intake in Tromsø4, Tromsø6 or Tromsø7’ (i.e. not at all). Thus, participants are of eligible age and with data on minimum one fish variable from any of the three surveys. ^c^Stable patterns of fish intake defined as the same reported frequency of intake in all three surveys, or two similar frequencies of intake plus one frequency of intake differing by one level. ^d^Reference category. ^e^Inconsistent patterns defined as patterns of fish intake that spread across the three levels of frequency of intake. **Model 1:** adjusted for Tromsø6 age and sex. **Model 2:** adjusted for Tromsø6 age, sex, BMI, education, comorbidity, smoking, activity level and self-reported health. **Model 3:** additionally adjusted for Tromsø6 cod liver oil and/or long-chain omega-3-fatty acids supplement use.

**Table S9** Onset of physical frailty characteristics in Tromsø7 (*n* = 4350)^a^

|  | **All**  **(*n* = 4350)** | **Frailty score 1**  **(*n* = 1031)** | **Frailty score 1–2**  **(*n* = 1224)^b^** |
| --- | --- | --- | --- |
| Frequency of frailty components (%)^c^  Exhaustion, *n* = 4139  Slow walking speed, *n* = 3182  Low grip strength, *n* = 3185  Weight loss, *n* = 4194  Low physical activity, *n* = 4012 | 2.9  5.4  6.3  7.3  15.5 | 7.8  13.7  17.6  23.3  51.4 | 10.5  19.3  22.1  26.1  54.3 |

^a^Main analytic sample. ^b^Pre-frail participants. ^c^Percentage prevalence calculated among participants with valid data on the specific frailty components (left column).

**Table S10** Frailty prevalence in Tromsø7 stratified by age (*n* = 4350)^a^

|  | **Robust**  **(*n* = 3126)** | **Pre-frail**  **(*n* = 1224)** | ***P*_trend_^b^** |
| --- | --- | --- | --- |
| Age (years) in Tromsø7 |  |  |  |
| 65–69, *n* = 1373 | 76.3 | 23.7 | <0.001 |
| 70–74, *n* = 1497 | 75.4 | 24.7 |  |
| 75–79, *n* = 843 | 67.1 | 32.9 |  |
| >80, *n* = 637 | 60.4 | 39.6 |  |

^a^Main analytic sample. ^b^*P* value: Cochran-Armitage test for trend across groups.
